# Development of a cost-effective, morphology-preserving method for DNA isolation from bulk invertebrate trap catches: Tephritid fruit flies as an exemplar

**DOI:** 10.1371/journal.pone.0281759

**Published:** 2023-02-15

**Authors:** Elizabeth V. Fowler, Melissa L. Starkie, Xiaocheng Zhu, Alexander M. Piper, Arati Agarwal, Lea Rako, Alexandra Gardiner, Sybilla Oczkowicz, David Gopurenko, Mark K. Schutze, Mark J. Blacket

**Affiliations:** 1 Queensland Department of Agriculture and Fisheries, Brisbane, QLD, Australia; 2 New South Wales Department of Primary Industries, Wagga Wagga Agricultural Institute, Wagga Wagga, NSW, Australia; 3 Agriculture Victoria Research, AgriBio Centre, Bundoora, VIC, Australia; United States Department of Agriculture, UNITED STATES

## Abstract

Insect identification and preservation of voucher specimens is integral to pest diagnostic and surveillance activities; yet bulk-trapped insects are a diagnostic challenge due to high catch numbers and the susceptibility of samples to environmental damage. Many insect trap catches rely on examination of morphological characters for species identifications, which is a time consuming and highly skilled task, hence there is a need for more efficient molecular approaches. Many bulk DNA extraction methods require destructive sampling of specimens, resulting in damaged, or fully destroyed, voucher specimens. We developed an inexpensive, rapid, bulk DNA isolation method that preserves specimens as pinned vouchers to a standard that allows for post-extraction morphological examination and inclusion in insect reference collections. Our protocol was validated using a group of insects that are time-consuming to identify when trapped in large numbers–the dacine fruit flies (Diptera: Tephritidae: Dacinae). In developing our method, we evaluated existing protocols against the following criteria: effect on morphology; suitability for large trap catches; cost; ease of handling; and application to downstream molecular diagnostic analyses such as real-time PCR and metabarcoding. We found that the optimum method for rapid isolation of DNA extraction was immersing flies in a NaOH:TE buffer at 75°C for 10 minutes, without the need for proteinase K or detergents. This HotSOAK method produced sufficient high-quality DNA whilst preserving morphological characters suitable for species-level identification with up to 20,000 flies in a sample. The lysates performed well in down-stream analyses such as loop-mediated isothermal amplification (LAMP) and real-time PCR applications, while for metabarcoding PCR the lysate required an additional column purification step. Development of this method is a key step required for upscaling our capacity to accurately detect insects captured in bulk traps, whether for biodiversity, biosecurity, or pest management objectives.

## Introduction

The use of traps to collect insects is a common practice employed to meet a wide range of objectives from biodiversity research to biosecurity surveillance [1,2]. Trapping methods are often tailored to a target species or group and take advantage of cues such as chemical stimulants, visual attractants, or both [2]. Regardless of the means, a common issue across trapping studies is the ‘diagnostic bottleneck’: potentially hundreds or thousands of insects caught in the span of a few days taking days or weeks to sort through and identify. The identification process is further delayed if diagnosticians are faced with decayed or structurally compromised samples. Whilst manually sorting traps and identifying specimens through microscopic examination is a time-consuming and expensive process for biodiversity studies, efficient and accurate diagnostics is critical for biosecurity surveillance operations where diagnostic results are required within very short timeframes.

Molecular genetic methods are often used to complement, and sometimes replace, morphological identifications of trapped samples. Where specimens are damaged or their identification is ambiguous (e.g., member of a cryptic species complex), species level genetic diagnostics is often achieved using either the 5’ cytochrome *c* oxidase subunit I (COI) gene region [3] or other taxon-specific diagnostic loci. Given the increased utility of genetic data to support species identification, there has been a shift from time-consuming morphological identification to bulk processing of trap catches for downstream genetic characterisation. Downstream approaches, such as metabarcoding identification and real-time quantitative PCR, have become increasingly popular for such bulk insect identification [4,5].

To maintain data integrity and reporting standards, diagnostic labs may be required to perform post-extraction morphological examination and, ideally, incorporate suspect insects into reference collections as voucher specimens [6]. Current non-destructive methods of DNA extraction commonly use expensive reagents [7]. Alternatively DNA extraction requires destructive sampling which does not preserve voucher specimens for verification or accessioning into collections [8]. In addition, to ensure all individuals are accurately diagnosed, extraction methods must yield enough high quality DNA for successful molecular analyses while retaining sufficient sensitivity to detect exotic species at very low frequencies [8,9].

The above said, not all morphological characters are equal for all species, and the effect of physical degradation following non-destructive DNA extraction may also differ depending on the species. For instance, some insects (e.g., beetles) may survive a wide range of non-destructive extraction techniques, leaving heavily sclerotised diagnostic features intact [10]. In contrast, for other insects their primary diagnostic features, such as integument colour, may be degraded by even the most carefully applied non-destructive techniques. Therefore, there is a need for further development of appropriate bulk non-destructive DNA extraction methods across a range of taxa where such sorting and identification is undertaken through traditional microscopic examination. This is especially needed for groups where colour pattern remains the defining character used for species delimitation, such as for dacine fruit flies [11].

Dacine fruit flies (Diptera: Tephritidae: Dacini) are a diverse and abundant group of insects targeted in evolutionary, biogeographic, and biodiversity research [12–14] but are perhaps most widely known as high-priority horticultural pests [15]. Primarily represented by genera *Bactrocera* Macquart, *Zeugodacus* Hendel, and *Dacus* F., the group consists of over 900 species [16,17] and their identification relies on a combination of colour patterns and structural characters. While identification using such characters may be readily achieved for distinctive species (e.g., the bread-fruit fly, *Bactrocera umbrosa*), for others it is problematic due of multiple morphologically cryptic species groups in the group, such as the Oriental fruit fly (*Bactrocera dorsalis*) complex which consists of several highly similar species that may be readily confused with each other [12]. Additionally, in cases where species are genetically inseparable using available markers, subtle variation in colour pattern is sometimes the only tool available for identification; yet see [18,19] where genetic inseparability and subtle morphological variation was deemed to represent intraspecific variation resulting is the synonymy of key pest taxa. Hence, preservation of specimen morphology during DNA extraction is essential for post-extraction confirmation of ambiguous samples.

Physical approaches to DNA extraction such as bead beating, grinding, or freeze thawing are not suitable as they are destructive methods that would cause structural damage and obliterate diagnostic characters. Alternative chemical-based methods use detergents, alkaline lysis or chaotropic agents to solubilise or denature cellular components [20] and permit the retention of specimens; however, application of chemical lysis to non-destructive (ND) DNA isolation using these agents is typically accompanied by the addition of proteinase K which adds cost to processing samples, especially if there are many. Several studies have developed non-destructive methods along these lines for insects as single specimens or small samples [6,10,21] as well as for bulk mixed species trap samples [22–25]. Morphology of arthropod samples was found to be well-preserved in [22] and the issue of bulk DNA extraction in a wide range of insects (malaise-trapped) while preserving specimens for post-extraction taxonomic work was further addressed by Kirse and co-workers [24]; however, as alluded to above, both of these studies lysed cells using relatively expensive proteinase K and sodium dodecyl sulphate (SDS). Simple buffer-based methods, which do not rely upon proteinase K for cell lysis, have been developed [26,27], have been applied to bulk invertebrate samples [8] and represent promising avenues for further optimisation.

A further study of three non-destructive methods commonly used for entomophagous insects found that the alkaline lysis method known as “HotSHOT” performed comparably well to two commonly used ND methods (modified Qiagen DNeasy® Blood & Tissue kit; modified calcium chloride lysis), with little effect on morphology [28], suggesting this method may be suitable for broader application across other insects. A modified version of this buffer, called “HotSHOT 6", was developed for use in mussels [26] and has recently been applied to non-destructive DNA isolation from several pest insect species [21,29,30]. The application of this method to bulk insect samples would significantly reduce labour requirements for rapid diagnosis of large trap catches, however it has yet to be evaluated for this purpose.

Despite the success of these bulk extraction approaches for other taxa, we do not consider them to meet all of our criteria for bulk processing of dacine fruit flies as published, specifically: i) DNA quality and quantity; ii) morphological preservation (esp. colour); iii) reduced cost through elimination of expensive reagents (esp. proteinase K and lysis buffer). The last consideration is especially pertinent for extensive surveillance programmes such as undertaken in Queensland, as proteinase K and commercial lysis buffers may add significant cost in time and resources when processing a significant number of traps where up to 30,000 flies may be caught in a single trap (unpubl. data, Northern Australian Quarantine Survey). We therefore aimed to focus on two of the most promising approaches [21,22,26] to optimise for the tephritid fruit fly bulk surveillance trap catches.

Taking these considerations into account, our aims were to develop a non-destructive extraction protocol that: (1) had minimal impact on morphology, especially colour, so that voucher specimens can be validated and/or preserved; (2) was cost effective and simple by minimising the use of, or eliminating, reagents such as proteinase K or detergents and is potentially suitable for infield use; (3) uses non-toxic reagents (4); produces high DNA quality and yield; (5) has the capacity to be scaled up for bulk samples; (6) produces extracts that are suitable for use in downstream molecular diagnostic applications (i.e., loop-mediated isothermal amplification [LAMP], real-time PCR and metabarcoding); and finally, (7) was applicable to our chosen study group, the dacine fruit flies, as a means to evaluate the potential for bulk molecular screening of fruit fly trap samples to support Australian biosecurity surveillance and diagnostics operations. We anticipate that successful bulk DNA extraction whilst preserving tephritid fruit fly morphology will translate to other insect groups where diagnostics characters, especially colour, must be preserved.

## Results

Two buffer solutions were evaluated in this study: HotSOAK Buffer 1 (modified HotShot protocol, HS6, from [21,26]) and HotSOAK Buffer 2 (modified from [22]). The effect of adding a detergent was also tested in both buffers. A five-point scale was developed to classify the damage caused by these buffers on fruit fly morphology (i.e., 1–2 = too damaged to identify; 3–4 = some damage, but identifiable; 5 = undamaged) (refer to methods for more detail). Temperature and incubation time required for good quality DNA yield was also evaluated and a final rapid method suitable for bulk sample processing was developed.

### Detergent selection

DNA quality and yield was highest for both the Buffer 1 and Buffer 2 lysis buffer with the addition of 2% sodium dodecyl sulphate (SDS) compared to all combinations tested (Table 1). The addition of Triton-X-100 (Tx100) had a strong negative effect on DNA yield, independent of lysis buffer, with little to no DNA present in the Buffer 1 and Buffer 2 treatments, respectively.

**Table 1:** Purity and yield of DNA extracts and morphology following lysis of approx. 0.1 g fruit flies (10–15) at 56°C overnight in 1mL of either Buffer 1 or Buffer 2 containing Tx100 or SDS at 2%. BD = below detection. Ct = cycles above threshold (threshold set at 0.05).

Buffer	Morphology score*	Concentration (ng/μL)	18S real-time PCR (Ct value)	A260/280 ratio
Buffer 1 + 2% SDS	3	29.9	13.7	2.1
Buffer 1 + 2% Tx100	3	3.2	19.9	1.5
Buffer 2 + 2% SDS	4	3.4	21.1	1.6
Buffer 2 + 2% Tx100	4	BD	BD	0.7

* 3 = usually identifiable; 4 = always identifiable.

All DNA extracts, except Buffer 2 with Tx100, were amplified in the 18S real-time PCR assay, and the cycle threshold (Ct) values produced by DNA extracted from flies treated with 2% SDS in either Buffer 1 or Buffer 2 are indicative of good quality DNA. The effects of lysis treatment on morphology were more detrimental in Buffer 1 compared with Buffer 2 overall with scores of 3 and 4, respectively (Table 1). We discontinued the use of Tx100 as an additive detergent and only used SDS for further downstream optimisation. Both buffers were tested further because Buffer 1 yielded more DNA while Buffer 2 appeared better at preserving morphological features for species identification.

### Effect of SDS concentration and buffer selection

Congruent with our initial results, Buffer 2 treatments yielded lower DNA than Buffer 1 treatments, particularly when used with lower SDS concentrations. There was an increase in DNA yield with higher SDS concentration in Buffer 2, while the opposite was observed with Buffer 1 (Table 2). Absorbance ratios showed no obvious relationship with change in SDS concentration and DNA quality. All DNA extracts were amplified in the 18S real-time PCR with 0.5–1.0% SDS treatments producing similarly lower Ct values for both buffers. The effect of SDS concentration on morphology in these two buffers was different and showed some variability, particularly in Buffer 2. When SDS concentration in Buffer 1 was increased to > 0.25% the morphology score decreased (i.e., increased damage/ loss of colour), while the addition of 0.25% SDS to Buffer 2 increased the morphology score (i.e., less damage) (Table 2). However, at higher concentrations of SDS (0.5–0.75%) in Buffer 2 morphology scores were lower. Considering these results, we decided to discontinue Buffer 2 from any further testing. We also discontinued with SDS as the addition of any amount > 0.25% was found to be detrimental to morphology, and there was no appreciable improvement in DNA quality or yield with the addition of 0.25% SDS compared with Buffer 1 only.

**Table 2:** Evaluation of Buffer 1 and Buffer 2 with 0 to 1% SDS lysis treatments of approx. 0.7 g dacini fruit flies on morphology and DNA extract purity and yield (BD = below detection; Ct = cycles above threshold).

Buffer	Morphology Score*	Concentration (ng/μL)	18S real-time PCR (Ct value)	A260/280 ratio
Buffer 1 only	4	15.0	25.3	1.8
Buffer 1 + 0.25% SDS	4	17.0	28.7	2.2
Buffer 1 + 0.5% SDS	2	13.4	17.0	2.1
Buffer 1 + 0.75% SDS	2	11.4	15.3	2.1
Buffer 1 + 1% SDS	3	11.1	15.6	2.1
Buffer 2 only	2	2.8	33.8	1.5
Buffer 2 + 0.25% SDS	4	BD	23.4	2.3
Buffer 2 + 0.5% SDS	3	3.0	19.2	1.9
Buffer 2 + 0.75% SDS	2	10.0	16.5	2.0
Buffer 2 + 1% SDS	4	9.3	17.8	2.0

*2 = not identifiable; 3 = usually identifiable; 4 = always identifiable.

### Development of rapid method and bulk extraction trial

Overall, incubation of 0.7 g (approx. 100) flies in 5 mL of Buffer 1 at 75°C for 10 minutes yielded a higher concentration of DNA compared to 56°C for 10 minutes (Table 3). The morphology score was 4 for both groups, however the DNA yield (Qubit) was double at the higher temperature and was below detection in 18S real-time PCR for the lower temperature (Table 3). Pre-heating buffer to 75°C prior to incubation yielded similar DNA results to unheated, while results were similar from bulk samples when 16.5 g flies were lysed in 50 mL of Buffer 1 for 10 minutes (Table 3). We also observed that the Ct values were consistently <16–17 for both small scale and bulk rapid 75°C isolations, compared with the overnight Buffer 1 only 56°C isolation (Table 3) (Ct >25).

**Table 3:** Evaluation of rapid lysis method (i.e., 10-minute incubation at increased temperature) on fruit fly morphology, DNA yield and quality (ND = No Data; BD = below detection).

Incubation Temperature	Morphology Score*	Concentration (ng/μL)	18S real-time PCR (Ct value)	A260/ 280 ratio
56°C	4	2.8	BD	2.0
75°C^#^	4	4.6 ± 1.8	17.8 ± 2.0	2.3
75°C preheated	4	4.9	17.8	ND
Bulk 75°C^#^	4	4.7 ± 0.0	16.1 ± 0.3	2.3

*4 = always identifiable.

^**#**^Duplicate treatments tested–data shown is average ± standard deviation.

When evaluating effect of cold storage duration and temperature on DNA yield and quality, there was an increase in Ct value of 1.9 for the Buffer 1 lysates stored for one month at 4°C compared to fresh Buffer 1 lysates, while there was very little change between the fresh and frozen samples (< 0.3 Ct increase) (see S1 Table).

### Increasing incubation time

**Fig 1:**
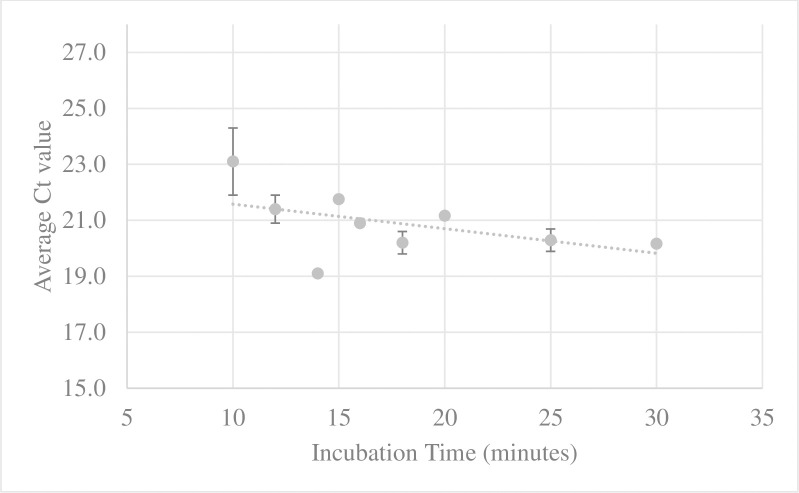
Effect of Buffer 1 incubation time on template yield measured by 18S rRNA real-time PCR (average Ct value; error bars represent the standard deviation between Ct values).

We found that increased incubation times produced more DNA, with 18S rRNA real-time PCR Ct values reducing 2.9 cycles (23.1 to 20.2 cycles) from 10 to 30 minutes, with the greatest Ct decrease observed at 14 minutes (3.0 cycles) (Fig 1; S2 Table). In addition, after 14 minutes of incubation, the morphology began to significantly decline in quality, and by 16 minutes flies were deemed unidentifiable as they were dark with dulled colours and structural damage, i.e., morphology score of ≤ 2. In our first time-course experiment, we collected serial lysate aliquots from the one pot of flies, thus reducing the volume over the course of the treatment, which may have affected our DNA yields. However, the subsequent treatments of 100 and 300 flies for 10 minutes and 20 minutes showed little difference in the Ct values between these two timepoints for the 18SrRNA real-time PCR (Ct difference = 0.7–0.9). There was a 4 to 6-fold increase in DNA yield when lysis time was increased; however, this the corresponding Ct values showed minimal decrease relative to this (relative Ct decrease = 0.3–0.9). Based on this, we decided to use the 10 minutes and 20 minutes incubation times for testing with downstream applications.

### Application to downstream molecular methods

All lysates and pure DNA extracts tested in LAMP and real-time PCRs produced measurable results with little variation seen between samples from 100 or 300 flies, incubated for 10 or 20 minutes (Real-time PCR Ct values for *B*. *tryoni* = 19.9–22.2 and 18S rRNA = 17.7–21.5; S3 Table). The only variability in amplification observed between purified DNA extracts and crude lysates was with the metabarcoding PCR. Weak products or no amplification was observed for the lysates with this PCR, while pure DNA extracts amplified sufficient product for further downstream metabarcoding processes. A single *B*. *jarvisi* spiked into 100–300 *B*. *tryoni* was detected by real-time PCR amplification from both DNA extracts and HotSOAK lysates, with lower Ct values (ie. higher concentrations) in the 100 flies, however little difference observed between the crude lysates and the pure DNA extracts (see S3 Table). This optimised method was tested in bulk samples of up to 140 g (approx. 20,000 by weight) flies spiked with one *B*. *jarvisi* and five *Z*. *cucumis*. Using species-specific real-time PCR, we were able to consistently detect a single fly in bulk samples of up to 63 g flies (approx. 9,000 by weight). In the larger 140 g sample we were still able to detect the spiked-in fly species, but only in 60–70% of the lysate aliquots sampled (see S4 Table).

## Discussion

We developed an inexpensive and rapid “HotSOAK” method for isolation of high-quality DNA from bulk fruit fly samples. Our method is cost-effective, utilises easily acquired reagents, and preserves samples for follow-up morphological examination. This is particularly critical for morphological identification of dacine fruit flies, which relies on a combination of colour patterns and structural characters [31]. This is the first time that a method has successfully been developed to rapidly isolate good quality DNA from bulk insect samples without the need for enzymes or detergents to facilitate release from cells, while preserving specimens for post-extraction taxonomic work.

Fruit fly morphological characters are extremely susceptible to damage and discolouration. During our trials, we observed overall darkening in colour, with discolouration and fading occurring in some samples. Flies treated with our HotSOAK method tended to be better preserved when treated with Buffer 1 as compared to Buffer 2. Further to this, structural damage (esp. shrivelled eyes) was more apparent from flies subjected to Buffer 2 treatments, an effect either not seen or significantly diminished in those treated with HotSOAK Buffer 1. We note that eyes are not a diagnostic character for dacine tephritids, yet structural damage such as this may be critical for other insect taxa where these treatments are considered.

There are many other non-destructive methods available for DNA isolation from insects [6,10,22,23,32], but our study brings together several criteria that have not previously been considered collectively. Our method is rapid, cost-effective, suitable for bulk samples and is gentle on specimens. Crucially, we were able to isolate sufficient DNA for many downstream molecular applications without addition of proteinase K or chemical lysis reagents which are common components in many published non-destructive methods [22,33,34] and are cost-prohibitive for large scale bulk sample processing. Another consideration that we felt crucial was to reduce physical damage to specimens by decreasing the processing time and minimising sample handling. Some published methods involve piercing holes in each sample [32] which is laborious and potentially detrimental to specimen integrity. We were able to minimise loss of pigment in specimens such that colour patterns were preserved sufficiently for fruit fly species identification. We believe this is due to the very short amount of time that specimens were subject to treatment in the lysis buffer. Sample processing times of other non-destructive DNA isolation methods are much longer by comparison, taking anywhere from 2.5 hours up to 3 days for the lysis step [6,22–24,34]. In our study, incubation longer than 14 minutes was detrimental to morphology at 75°C, however overnight incubation at 56°C was not as damaging. For specimens where there is no immediate need for a result (i.e., biodiversity studies), samples could be incubated at a lower temperature for longer [34], but for surveillance samples, it would be an advantage to opt for short processing times at the higher temperature because of the fast turnaround required in diagnostics.

Our HotSOAK method is cost effective compared to commercially available kits. Many commercially available kits utilise proteinase K or detergents [35]. The average cost of these reagents can be upwards of AUD$1.50 per sample (for Proteinase K) and AUD$0.65/ mL (e.g., ATL lysis buffer). The reagents we identified as the components of Buffer 1, cost a total of AUD$0.01/ mL, and are therefore comparably cheaper to the methods outlined in [7], although we did find that a final column extraction was required for some downstream approaches (such as metabarcoding), which would add $4–6 per sample, however this cost could be reduced by using an in-house DNA extraction method (i.e. high salt method).

The HotSOAK method is a novel protocol, based on the HotSHOT 6 method developed for freshwater mussels [26]. The original HotSHOT method was first described as a two-step rapid alkaline lysis protocol for DNA isolation from mouse tissue over two decades ago [27], and was also found to be effective for insects both for sufficient DNA yield [36] and morphological preservation [28]. HotSHOT 6 is a modified rapid one-step method that combines the alkaline lysis and neutralisation buffers into one, with a single 20 minute incubation [26]. Three reports have utilised this HotSHOT 6 buffer, but with reduced incubation times to preserve specimens for subsequent morphological examination [21,29,30], however its application on bulk samples had not been previously considered. We modified the buffer volumes and incubation temperature to develop a method that could be scaled up for bulk sample processing.

Crude lysates extracted using the HotSOAK method were suitable for bulk processing of large trap catches. Lysates performed well in down-stream analyses such as loop-mediated isothermal amplification (LAMP) and real-time PCR applications, with a final column purification required for the metabarcoding PCR. We suspect that this was due to enzyme inhibitors or potential contaminants (i.e. MyFi Polymerase has a known enzyme sensitivity to the EDTA in TE buffer [37]). Additionally, there are numerous cellular constituents that can act as DNA polymerase inhibitors including proteins, polysaccharides, cell debris and exogenous DNA [38]. Further work would be required to optimise this reaction or trial alternative enzymes that are resistant to PCR inhibition such as those recently reported by Stein and co-workers [39]. Alternatively, reducing the EDTA concentration in Buffer 1 could also be trialled, although this is already relatively low and may affect the long-term preservation of isolated DNA in crude preparations.

Lastly, a final caveat we wish to raise is that our method development was undertaken under a relatively narrow band of ideal laboratory conditions, especially in the use of colony-reared flies towards method development. Tephritid (and other insect) trapping approaches may vary greatly in terms of preservation method (dry, ethanol, or propylene glycol), length of placement in field (from days to weeks), and local environmental conditions (dry to humid; cold to hot), and we did not seek to explicitly and evaluate each of these potential variables and their effect on fly DNA quality and quantity or morphological preservation using the HotSOAK approach. Future research in this approach will evaluate the technique for a wider range of tephritid trap catches and we recommend potential users of this approach to evaluate it under their specific circumstances prior to widespread deployment.

## Conclusions

There are numerous applications for this morphology-preserving DNA isolation method. We have tailored our method to best suit fruit flies, however, this method may be applied and modified to suit any invertebrate species, particularly taxa where morphological preservation is crucial for subsequent validation or vouchering purposes. Development of this HotSOAK method is a key step towards enhancing our capacity to accurately detect insects captured in bulk traps, whether for biodiversity or biosecurity objectives.

## Materials and methods

### Origin of samples

A combination of wild-caught and colony-reared fruit flies were used in method evaluation. Wild-caught flies were obtained from traps maintained by Biosecurity Queensland (Queensland Department of Agriculture and Fisheries [QDAF]) as part of the Exotic fruit fly surveillance program, which were cleared between August and September 2020. Colony flies were sourced from *B*. *tryoni* (Froggatt), *B*. *neohumeralis* (Hardy), *B*. *jarvisi* (Tryon), *B*. *kraussi* (Hardy) and *B*. *bryoniae* (Tryon) colonies maintained by the QDAF in Brisbane and Cairns; *B*. *tryoni* and *Zeugodacus cucumis* (French) colonies maintained by NSW DPI in Qurimbah; as well as from a *B*. *tryoni* colony maintained by AgVic in Melbourne. Due to limitations in availability of sufficient fruit flies to make up mock bulk samples for the 1:20,000 trial, soft-bodied flies of similar size and weight were sourced from *Musca domestica* (Linnaeus), *Lucilia cuprina* (Wiedemann) and *Chrysomya megacephala* (Fabricius) colonies maintained by QDAF in Brisbane. All insects were reared to adults and killed by freezing at -20°C.

### Morphological assessment of flies post-extraction

**Fig 2:**
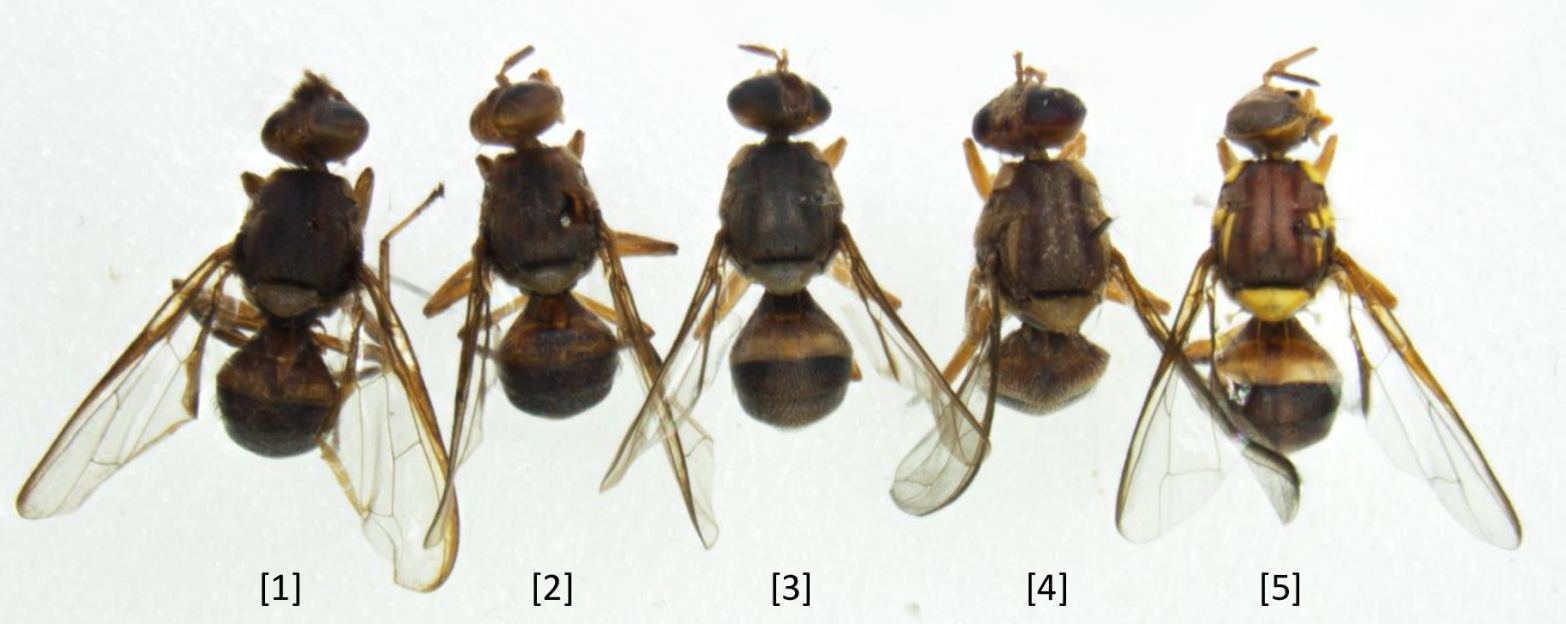
Examples of numerical scale used in scoring damage caused to dacine fruit fly characters by lysis buffers trialled in this study, using *B*. *tryoni* as an example. The fly on the far right-hand side was untreated, i.e., score 5, while the four flies to the left of this were treated resulting in damage scores of 1 to 4, in order from left to right.

Morphological evaluation following DNA extraction was carried out by eye using a Leica M80 stereo microscope to assess the visibility of morphological characters, pre- and post-treatment. Extraction methods that yielded samples with physical damage were noted and ranked lower (e.g., shrivelling of the eyes and head), as were those with notable discolouration to diagnostic features (e.g., vittae, post-pronotal lobes and scutellum). The following numerical scale was developed to score the effects of lysis treatments on morphology: (1) unidentifiable; (2) dark, structures damaged and difficult to see; (3) colours dull, some damage or bright colours lacking; (4) colours dull but no damage; (5) colours and structures intact, i.e., pristine. Flies with a score below 3 could not be reliably identified. Flies with a score of 3 were usually identifiable depending on the effect on colour. Flies with a score of 4 and above could always be identified and were considered suitable for accession into a collection (see Fig 2 for examples of scoring). Flies with a score of 5 were restricted to those that had undergone no DNA extraction at all (i.e., freshly killed and in perfect condition); therefore, no DNA-extracted specimens for any of the experiments scored a ‘5’ as there was always some impact on morphology (especially colour).

### Buffer solutions tested

Two buffer solutions were evaluated in this study: HotSOAK Buffer 1 (modified HotShot protocol, HS6, from [21,26] consisted of 5 mmol/L Tris-HCl (pH8.0), 0.5 mmol/L EDTA (pH 8.0), 12.5 mmol/L NaOH in sterile milli-Q water; and HotSOAK Buffer 2 (modified from [22], which consisted of 10 mmol/L Tris-HCl (pH8.0), 10 mmol/L NaCl, 5 mmol/L CaCl_2,_ 2.5 mmol/L ethylene‐diamine‐tetra‐acetic acid (EDTA) (pH 8.0) in sterile milli-Q water. Buffers were prepared from concentrated stock reagents made in-house or sourced commercially (Astral Scientific; Invitrogen, Australia).

For each experimental treatment, samples were mixed gently by inverting several times, 1.0 mL of lysis buffer (fly lysate) was transferred to a new 2.0 mL screw cap tube and stored overnight at 4°C for further DNA analysis. Flies were rinsed in absolute ethanol, strained, spread out evenly onto a 140 mm petri dish lined with KimWipes (Kimberley-Clark, Roswell, GA, USA) and for two hours at 40°C in a Clayson IM1000 incubator. Once dry, flies were sealed in a plastic container and stored at -20°C for morphological evaluation. The effects of both buffers were evaluated for impact on fruit fly diagnostic morphology and scored accordingly across a scale of 1–5 (Fig 2).

### Analysis of fly lysates

To evaluate DNA yield and quality, DNA was extracted from a 200–300 μL aliquot of lysate using the DNeasy® Blood and Tissue Kit (Qiagen, Germany) following the Animal Blood protocol without proteinase K digestion. Our modifications to the protocol were: omitting step 1 of the standard protocol and the 10 minutes incubation at 56°C; and eluting into 50–75 μL of elution buffer. Extracted DNA yield and quality were evaluated using three approaches: i) DNA concentration measurements taken on a Qubit® 2.0 fluorometer (Invitrogen, Carlsbad, CA, USA); ii) Absorbance (260:280) ratio measurements taken on a Multiskan SkyHigh Microplate Spectrophotometer (Thermo Scientific^TM^, Waltham, MA, USA); iii) test in a real-time PCR using the commercial 18S rRNA probe-primer set (Applied Biosystems^TM^, Thermo Fisher Scientific, Australia), or the published *B*. *tryoni* COI assay [40,41], on a Rotor-Gene® Q real-time PCR system (Qiagen, Australia); real-time PCR probe and primer sequences, concentrations, reagents and reaction conditions are provided in the supporting information (S1 File). Lysates were also directly tested in real-time PCR.

### Detergent types and concentrations tested

We tested the effects of two detergent types on the lysis process for each of the Buffers: sodium dodecyl sulphate (SDS; Sigma; CAS #151-21-3) or Triton-X-100 (Tx100; Sigma; CAS #9036-19-5). Approximately 0.1 g of wild-trapped flies were submerged in 1 mL of each respective buffer and incubated in a 2 mL microfuge tube at 56°C overnight. We tested both Buffer 1 and Buffer 2 with the addition of 2% of Tx100 or SDS, after which we measured DNA yield and quality as well as morphological (primarily colour) preservation.

After determining the best-performing detergent based on DNA quality/quantity and morphological preservation, we sequentially reduced the detergent concentration (1%, 0.75%, 0.5%, 0.25%) to determine the minimal limit that met our quality criteria. We also scaled up the quantity of flies and volume of buffer proportionately; this time using 0.7 g wild-trapped flies (approx. 100–150 individuals) in 5 mL of each respective buffer in a 20 mL sterile container incubated overnight at 56°C. DNA yield, quality and morphology preservation were assessed as above.

### Development of HotSOAK rapid method and bulk lysis trial

Following determination of optimum buffer (Buffer 1, see results) and detergent concentration, our next step was to develop this method into a rapid approach suitable for high throughput, with a focus on repeatability and practicality for downstream applications, including use of lysates in LAMP, real-time PCR assays, and the generation of PCR amplicons for metabarcoding library preparation.

First, we evaluated the effect of reduced incubation time and higher temperature on the initial lysis step. Previous protocols using Buffer 1 used incubation times within a range of five to 20 minutes [21,26] incubated at 56°C; for this experiment we chose 10 minutes as our starting point as it was mid-way in this range, together with an elevated temperature of 75–80°C, based on the upper stable temperature limit of the water bath (n.b., while the set temperature of the water bath for testing was 80°C, we found the actual temperature of the lysates was 75°C; hence reported as the minimal temperature hereon). We used 0.7 g of colony flies in 5 mL of Buffer 1 across two treatments: 56°C for 10 minutes and 75°C for 10 min. Treatments were tested in duplicate and each lysate analysed in triplicate.

We further investigated the effects of incubation time on larger samples of flies (i.e., reflective of larger trap catches) in two steps. First, we extracted DNA from approx. 500 (3.5 g) colony flies in 10 mL of Buffer 1 at 75°C taking 0.5mL lysate aliquots at 10, 15, 20, 25 and 30 minutes (from one pot of flies). In this step our goal was to determine the effect of incubation time on DNA yield and morphology was examined at 30 minutes only. The second step focused on morphology samples at incubation times of 10, 12, 14, 16 and 18 minutes, from 3.5 g (approx. 500) colony flies in 10 mL of Buffer 1 at 75°C, and lysate samples were also collected at each time point. In this step, our goal was to identify the optimal incubation time for morphology preservation while not compromising DNA yield. Due to limited supplies of colony flies for this experiment we used *B*. *kraussi* for the first experiment and *B*. *tryoni* for the second. Here, our measurements of yield and quality were 18S rRNA real-time PCR on lysates (in triplicate) and morphological assessment.

The effect of pre-heating buffer to 75°C prior to incubation was also tested measuring DNA yield and quality by Qubit and *B*. *tryoni* COI assay real-time PCR. The final rapid non-destructive protocol was then tested on two bulk samples of flies where weight was used to estimate sample size (i.e., 3.5 g or approx. 500 flies). Here we tested 7.0 g (approx. 1000), 14.0 g (approx. 2000), 63 g (approx. 9000) and 140 g (approx. 20,000) flies, and 10ml of Buffer 1 was added/ 3.5 g of flies and lysed at 75°C for 10 minutes. Crude lysates (1ml) were collected in triplicate (five replicates for 140g sample) and tested in the species-specific real-time PCRs in triplicate for *B*. *jarvisi* and *Z*. *cucumis* real-time PCR (Li et al., 2019) (S1 File).

### Evaluation of HotSOAK protocol for downstream applications

The refined HotSOAK method developed from the above process was evaluated in downstream molecular applications using LAMP, species specific real-time PCR, and DNA metabarcoding PCR in a separate laboratory to where the method was developed (i.e., AgriBio Centre, Bundoora, Victoria). Colony flies (one *B*. *jarvisi* in a bulk sample of either 99 or 299 *B*. *tryoni*) were submerged in Buffer 1 and incubated at 75°C for 10 minutes as per the optimised protocol. DNA was extracted from an aliquot of lysate and quantified by Qubit. Crude lysates and DNA extracts were tested in the *B*. *tryoni* LAMP assay [40], *B*. *tryoni* real-time PCR, *B*. *jarvisi* real-time PCR [42] and 18S rRNA real-time PCR (Applied Biosystems^TM^, Thermo Fisher Scientific, Australia) (S1 File). Lysates and extracted DNA were tested for suitability in DNA metabarcoding using published PCR primers (fwhF2-fwhR2n) [43] and protocols [25] with 2.5 μL template at 2.5 ng/μL using MyFi HS DNA Polymerase (Meridian Bioscience Inc, Australia).

### Effect of cold storage on DNA yield and quality

We investigated the optimum of three storage methods for Buffer 1 lysates generated from extracted *B*. *tryoni* colony flies (3.5 g or approx. 500 flies). Lysates were tested in triplicate using the *B*. *tryoni* COI real-time PCR assay immediately after treatment to determine the quality and yield of the sample prior to storge. Three 200 μL aliquots of each treatment were collected, and stored at 4°C, -20°C and -80°C for four weeks. Following this, samples were removed from cold storage, thawed, and tested again in triplicate using the *B*. *tryoni* COI real-time PCR assay to assess loss in quality/degradation during storage at each temperature.

## Supporting information

S1 Table Effect of cold storage on DNA quality and yield from fresh lysates compared with lysates stored for one month at 4°C, -20°C or -80°C as measured by *B*. *tryoni* real-time PCR. All lysates were tested in triplicate.
(DOCX)

S2 Table Evaluation of increased incubation time on DNA quality in lysates for *Bactrocera* sp. colony flies (3.5g) lysed in HotSOAK Buffer 1 using 18S rRNA Real-time PCR. All lysates were collected and tested in triplicate.
(DOCX)

S3 Table Analysis of DNA isolated from 100 and 300 flies (one *B*. *jarvisi* made up to total with *B*. *tryoni*) treated with the optimised HotSOAK non-destructive method comparing crude lysate DNA and pure column-extracted DNA using QuBit DNA quantification, metabarcoding PCR, *B*. *tryoni* LAMP, and real-time PCR (*B*. *tryoni*, *B*. *jarvisi*, 18S rRNA). (DOCX)

S4 Table Real-time PCR detection of low frequency spiked-in fruit flies (one *Bactrocera jarvisi* and five *Zeugodacus* cucumis) in bulk samples of varying sizes (approximating 1000 to 20,000 fruit flies) extracted using the optimised HotSOAK method (10ml of Buffer 1 was added/ ~3.5 g of flies and lysed at 75°C for 10 minutes). Crude lysates and DNA extracts were tested in the species-specific real-time PCRs in triplicate for *Z*. *cucumis* and *B*. *jarvisi* real-time PCR (Li et al., 2019) (see above for method).
(DOCX)

S1 File Supplemental methods. (DOCX)

## References

[1] Cowley JM. A new system of fruit fly surveillance trapping in New Zealand. New Zealand Entomologist. 1990; 13(1):81–4.

[2] Tan KH, Nishida R, Jang EB, Shelly TE. Pheromones, Male Lures, and Trapping of Tephritid Fruit Flies. In: Shelly T, Epsky N, Jang EB, Reyes-Flores J, Vargas R, editors. Trapping and the Detection, Control, and Regulation of Tephritid Fruit Flies: Lures, Area-Wide Programs, and Trade Implications. Dordrecht: Springer Netherlands; 2014. p. 15–74.

[3] Hebert PD, Cywinska A, Ball SL, deWaard JR. Biological identifications through DNA barcodes. Proc Biol Sci. 2003; 270(1512):313–21. doi: 10.1098/rspb.2002.2218 12614582 PMC1691236

[4] Piper AM, Batovska J, Cogan NOI, Weiss J, Cunningham JP, Rodoni BC, et al. Prospects and challenges of implementing DNA metabarcoding for high-throughput insect surveillance. GigaScience. 2019; 8:1–22. doi: 10.1093/gigascience/giz092 31363753 PMC6667344

[5] Piper AM, Cunningham JP, Cogan NOI, Blacket MJ. DNA Metabarcoding Enables High-Throughput Detection of Spotted Wing Drosophila (Drosophila suzukii) Within Unsorted Trap Catches. Frontiers in Ecology and Evolution. 2022; 10.

[6] Martoni F, Valenzuela I, Blacket MJ. Non-destructive DNA extractions from fly larvae (Diptera: Muscidae) enable molecular identification of species and enhance morphological features. Austral Entomology. 2019; 58(4):848–56.

[7] Batovska J, Piper A, Valenzuela I, Cunningham J, Blacket MJ. Developing a non-destructive metabarcoding protocol for detection of pest insects in bulk trap catches. Scientific Reports. 2021; 11:7946. doi: 10.1038/s41598-021-85855-6 33846382 PMC8041782

[8] Majaneva M, Diserud OH, Eagle SHC, Hajibabaei M, Ekrem T. Choice of DNA extraction method affects DNA metabarcoding of unsorted invertebrate bulk samples. Metabarcoding & Metagenomics. 2018; 2:1–12.

[9] Martoni F, Nogarotto E, Piper AM, Mann R, Valenzuela I, Eow L, et al. Propylene Glycol and Non-Destructive DNA Extractions Enable Preservation and Isolation of Insect and Hosted Bacterial DNA. Agriculture. 2021; 11(1):77.

[10] Gilbert MTP, Moore W, Melchior L, Worobey M. DNA Extraction from Dry Museum Beetles without Conferring External Morphological Damage. PLOS ONE. 2007; 2(3):e272. doi: 10.1371/journal.pone.0000272 17342206 PMC1803022

[11] Plant Health Australia. Fruit Fly ID Australia 2018 [Available from: http://fruitflyidentification.org.au/.

[12] Clarke AR, Armstrong KF, Carmichael AE, Milne JR, Raghu S, Roderick GK, et al. INVASIVE PHYTOPHAGOUS PESTS ARISING THROUGH A RECENT TROPICAL EVOLUTIONARY RADIATION: The Bactrocera dorsalis Complex of Fruit Flies. Annual Review of Entomology. 2005; 50(1):293–319. doi: 10.1146/annurev.ento.50.071803.130428 15355242

[13] Novotny V, Clarke AR, Drew RAI, Balagawi S, Clifford B. Host specialization and species richness of fruit flies (Diptera: Tephritidae) in a New Guinea rain forest. Journal of Tropical Ecology. 2005; 21(1):67–77.

[14] Aluja M, Norrbom A. Fruit Flies (Tephritidae): Phylogeny and Evolution of Behavior. 1st Edition ed: CRC Press; 2001.

[15] White IM, Elson-Harris MM. Fruit flies of economic significance: their identification and bionomics. Wallingford, UK: CABI International; 1992.

[16] Doorenweerd C, Leblanc L, Norrbom AL, San Jose M, Rubinoff D. A global checklist of the 932 fruit fly species in the tribe Dacini (Diptera, Tephritidae). Zookeys. 2018; 730:19–56. 10.3897/zookeys.730.21786 PMC5799784 29416395

[17] Australian Faunal Directory [Internet]. Australian Biological Resources Study. 2020 [cited 19th July 2022]. Available from: https://biodiversity.org.au/afd/home.

[18] Doorenweerd C, San Jose M, Geib S, Dupuis J, Leblanc L, Barr N, et al. A phylogenomic approach to species delimitation in the mango fruit fly (Bactrocera frauenfeldi) complex: A new synonym of an important pest species with variable morphotypes (Diptera: Tephritidae). Systematic Entomology. n/a(n/a).

[19] SCHUTZE MK, AKETARAWONG N, AMORNSAK W, ARMSTRONG KF, AUGUSTINOS AA, BARR N, et al. Synonymization of key pest species within the Bactrocera dorsalis species complex (Diptera: Tephritidae): taxonomic changes based on a review of 20 years of integrative morphological, molecular, cytogenetic, behavioural and chemoecological data. Systematic Entomology. 2015; 40(2):456–71.

[20] Lever MA, Torti A, Eickenbusch P, Michaud AB, Santl-Temkiv T, Jorgensen BB. A modular method for the extraction of DNA and RNA, and the separation of DNA pools from diverse environmental sample types. Frontiers in Microbiology. 2015; 6:476. doi: 10.3389/fmicb.2015.00476 26042110 PMC4436928

[21] Agarwal A, Cunningham JP, Valenzuela I, Blacket MJ. A diagnostic LAMP assay for the destructive grapevine insect pest, phylloxera (*Daktulosphaira vitifoliae*). Scientific Reports. 2020; 10:21229. 33277555 10.1038/s41598-020-77928-9 PMC7718921

[22] Nielsen M, Gilbert MTP, Pape T, Bohmann K. A simplified DNA extraction protocol for unsorted bulk arthropod samples that maintains exoskeletal integrity. Environmental DNA. 2019; 1:144–54.

[23] Ritter CD, Haggqvist S, Karlsson D, Saaksjarvi IE, Muasya AM, Nilsson RH, et al. Biodiversity assessments in the 21st century: the potential of insect traps to complement environmental samples for estimating eukaryotic and prokaryotic diversity using high-throughput DNA metabarcoding. Genome. 2019; 62:147–59. 30673361 10.1139/gen-2018-0096

[24] Kirse A, Bourlat SJ, Langen K, Zapke B, Zizka VMA. Comparison of destructive and nondestructive DNA extraction methods for the metabarcoding of arthropod bulk samples. Molecular Ecology Resources. 2022; 00:1–14. doi: 10.1111/1755-0998.13694 35932285

[25] Martoni F, Piper AM, Rodoni BC, Blacket MJ. Disentangling bias for non-destructive insect metabarcoding. PeerJ. 2022; 10:e12981. doi: 10.7717/peerj.12981 35228909 PMC8881911

[26] Zieritz A, Yasaeng P, Razak NFA, Hongtrakul V, Kovitvadhi U, Kanchanaketu T. Development and evaluation of hotshot protocols for cost- and time-effective extraction of PCR-ready DNA from single freshwater mussel larvae (Bivalvia: Unionida). Journal of Molluscan Studies. 2018; 84:198–201.

[27] Truett GE, Heeger P, Mynatt RL, Truett AA, Walker JA, Warman ML. Preparation of PCR-quality mouse genomic DNA with hot sodium hydroxide and tris (HotSHOT). Biotechniques. 2000; 29(1):52, 4. doi: 10.2144/00291bm09 10907076

[28] Suaste-Dzul AP, Rodríguez-Vélez JM, Rodríguez-Vélez B, Arredondo-Bernal HC, Gallou A. Non-destructive DNA extraction methods for entomophagous insects with emphasis on biological control. Genome. 2019; 62(4):287–93. doi: 10.1139/gen-2018-0045 30817213

[29] Jiao J, Ren L, Chen R, Tao J, Luo Y. A LAMP Assay for the Detection of Thecodiplosis japonensis, an Alien Gall Midge Species Pest of Pine Trees. Insects. 2022; 13(6):540. doi: 10.3390/insects13060540 35735877 PMC9225623

[30] Rako L, Agarwal A, Semararo L, Broadley A, Rodoni BC, Blacket MJ. A LAMP (Loop-mediated isothermal amplification) test for rapid identification of Khapra beetle (*Trogoderma granarium*). Pest Management Science. 2021; 77:5509–21. 34363302 10.1002/ps.6591 PMC9290502

[31] Plant Health Australia. The Australian Handbook for the identification of fruit flies. Canberra, ACT: Plant Health Australia; 2018.

[32] Castalanelli MA, Severtson DL, Brumley CJ, Szito A, Foottit RG, Grimm M, et al. A rapid non-destructive DNA extraction method for insects and other arthropods. Journal of Asia-Pacific Entomology. 2010; 13(3):243–8.

[33] Miura K, Higashiura Y, Maeto K. Evaluation of easy, non-destructive methods of DNA extraction from minute insects. Applied Entomology and Zoology. 2017; 52(2):349–52.

[34] Marquina D, Roslin T, Lukasik P, Ronquist F. Evaluation of non-destructive DNA extraction protocols for insect metabarcoding: gentler and shorter is better. Metabarcoding & Metagenomics. 2022; 6:187–201.

[35] Psifidi A, Dovas CI, Bramis G, Lazou T, Russel CL, Arsenos G, et al. Comparison of eleven methods for genomic DNA extraction suitable for large-scale whole-genome genotyping and long-term DNA banking using blood samples. PLoS One. 2015; 10(1):e0115960. doi: 10.1371/journal.pone.0115960 25635817 PMC4312062

[36] Guzmán-Larralde AJ, Suaste-Dzul AP, Gallou A, Peña-Carrillo KI. DNA recovery from microhymenoptera using six non-destructive methodologies with considerations for subsequent preparation of museum slides. Genome. 2017; 60(1):85–91. doi: 10.1139/gen-2015-0172 27996299

[37] Meridian Bioscience. MyFi Mix—product manual 2020 [updated 2020. Available from: https://www.bioline.com/myfi-mix.html.

[38] Schrader C, Schielke A, Ellerbroek L, Johne R. PCR inhibitors–occurrence, properties and removal. Journal of Applied Microbiology. 2012; 113(5):1014–26. doi: 10.1111/j.1365-2672.2012.05384.x 22747964

[39] Stein F, Wagner S, Bräsicke N, Gailing O, Moura CCM, Götz M. A Non-Destructive High-Speed Procedure to Obtain DNA Barcodes from Soft-Bodied Insect Samples with a Focus on the Dipteran Section of Schizophora. Insects. 2022; 13(8). doi: 10.3390/insects13080679 36005305 PMC9409269

[40] Blacket MJ, Agarwal A, Zheng L, Cunningham JP, Britton D, Schneider I, et al. A LAMP assay for the detection of *Bactrocera tryoni* Queensland fruit fly (Diptera: Tephritidae). Scientific Reports. 2020; 10:9554. 32533005 10.1038/s41598-020-65715-5 PMC7293347

[41] Dhami MK, Gunawardana DN, Voice D, Kumarasinghe L. A real-time PCR toolbox for accurate identification of invasive fruit fly species. Journal of Applied Entomology. 2016; 140:536–52.

[42] Li D, Nair S, Anderson D, Doddala P, Gunawardana DN, George S. Real‐time PCR assays for rapid detection of *Zeugodacus cucumis* and *Bactrocera jarvisi* (Diptera: Tephritidae) for quarantine application. Journal of Applied Entomology. 2019; 143:155–63.

[43] Vamos EE, Elbrecht V, Leese F. Short COI markers for freshwater macroinvertebrate metabarcoding. Metabarcoding and Metagenomics. 2017; 1.

