# Supplementary material for: Development of a cost-effective, morphology-preserving method for DNA isolation from bulk invertebrate trap catches: Tephritid fruit flies as an exemplar
Source: PLoS One. 2023 Feb 15;18(2):e0281759. doi: 10.1371/journal.pone.0281759 (PMC9931127; doi:10.1371/journal.pone.0281759)
Supplement: S1 Table — All lysates were tested in triplicate. (DOCX) [file pone.0281759.s001.docx]

**S1 Table.** Effect of cold storage on DNA quality and yield from fresh lysates compared with lysates stored for one month at 4^o^C, -20^o^C or -80^o^C as measured by *B. tryoni* real-time PCR. All lysates were tested in triplicate.

| Storage conditions | *B. tryoni* real-time PCR  (Ave Ct value) | Increase in Ct during storage |
| --- | --- | --- |
| Fresh | 19.2 ± 0.04 | N/A |
| 1 month at 4 °C | 21.2 ± 0.12 | 1.9 |
| 1 month at -20 °C | 19.5 ± 0.07 | 0.3 |
| 1 month at -80 ^°^C | 19.3 ± 0.14 | 0.1 |
