# Supplementary material for: Development of a cost-effective, morphology-preserving method for DNA isolation from bulk invertebrate trap catches: Tephritid fruit flies as an exemplar
Source: PLoS One. 2023 Feb 15;18(2):e0281759. doi: 10.1371/journal.pone.0281759 (PMC9931127; doi:10.1371/journal.pone.0281759)
Supplement: S2 Table — All lysates were collected and tested in triplicate. (DOCX) [file pone.0281759.s002.docx]

**S2 Table.** Evaluation of increased incubation time on DNA quality in lysates for *Bactrocera* sp. colony flies (3.5g) lysed in HotSOAK Buffer 1 using 18S rRNA Real-time PCR. All lysates were collected and tested in triplicate.

| Incubation Time (min) | 18S real-time PCR (Ave Ct value) |
| --- | --- |
| 10 | 23.1 ± 1.2 |
| 12 | 21.4 ± 0.5 |
| 14 | 19.1 ± 0.1 |
| 15 | 21.8 ± 0.0 |
| 16 | 20.9 ± 0.1 |
| 18 | 20.2 ± 0.4 |
| 20 | 21.2 ± 0.0 |
| 25 | 20.3 ± 0.1 |
| 30 | 20.2 ± 0.2 |
