# Supplementary material for: Development of a cost-effective, morphology-preserving method for DNA isolation from bulk invertebrate trap catches: Tephritid fruit flies as an exemplar
Source: PLoS One. 2023 Feb 15;18(2):e0281759. doi: 10.1371/journal.pone.0281759 (PMC9931127; doi:10.1371/journal.pone.0281759)
Supplement: S3 Table — (DOCX) [file pone.0281759.s003.docx]

**S3 Table.** Analysis of DNA isolated from 100 and 300 flies (one *B. jarvisi* made up to total with *B. tryoni*) treated with the optimised HotSOAK non-destructive method comparing crude lysate DNA and pure column-extracted DNA using QuBit DNA quantification, metabarcoding PCR, *B. tryoni* LAMP, and real-time PCR (*B. tryoni*, *B. jarvisi*, 18S rRNA).

|  |  |  |  | | ***B. tryoni* LAMP** | | **Real-time PCR (Ct value)** | | |
| --- | --- | --- | --- | --- | --- | --- | --- | --- | --- |
| **Sample** | **Incubation**  **Time (min)** | **DNA**  **concentration**  **(ng/µL)** | **Metabarcoding**  **PCR** | | **Amplification**  **Time**  **(min)** | **Melting**  **temp (**°C**)** | ***B. tryoni*** | ***B. jarvisi*** | **18S rRNA** |
| **Lysate DNA** |  |  |  |  |  |  |  |  |  |
| 100 flies | 10 |  | + | | 10.50 | 81.9 | 21.6 | 32.6 | 21.4 |
| (99 B.try+1 B.jar) | 20 |  | - | | 10.75 | 82.1 | 21.0 | 26.5 | 20.6 |
| 300 flies | 10 |  | + | | 11.00 | 82.0 | 22.2 | 34.5 | 21.5 |
| (299 B.try+1 B.jar) | 20 |  | - | | 11.25 | 82.0 | 21.4 | 32.7 | 20.6 |
|  |  |  |  |  |  |  |  |  |  |
| **Pure DNA** |  |  |  |  |  |  |  |  |  |
| 100 flies | 10 | 3.3 | + | | 8.75 | 81.8 | 20.3 | 31.5 | 19.2 |
| (99 B.try+1 B.jar) | 20 | 7.8 | + | | 8.75 | 82.0 | 19.9 | 25.6 | 18.3 |
| 300 flies | 10 | 19.9 | + | | 9.25 | 82.2 | 20.6 | 33.4 | 18.0 |
| (299 B.try+1 B.jar) | 20 | 33.3 | + | | 9.50 | 82.3 | 20.0 | 32.0 | 17.7 |
