# Supplementary material for: Development of a cost-effective, morphology-preserving method for DNA isolation from bulk invertebrate trap catches: Tephritid fruit flies as an exemplar
Source: PLoS One. 2023 Feb 15;18(2):e0281759. doi: 10.1371/journal.pone.0281759 (PMC9931127; doi:10.1371/journal.pone.0281759)
Supplement: S4 Table — Crude lysates and DNA extracts were tested in the species-specific real-time PCRs in triplicate for Z. cucumis and B. jarvisi real-time PCR (Li et al., 2019) (see above for method). (DOCX) [file pone.0281759.s004.docx]

| **Sample details** | | ***B. jarvisi* real-time PCR** | | | ***Z. cucumis* real-time PCR** | | |
| --- | --- | --- | --- | --- | --- | --- | --- |
| **weight (g)** | **Approx. # flies** | **Species detected** | **Ct Value Range** | **Ave. Ct value** | **Species detected** | **Ct Value Range** | **Ave. Ct value** |
| 3.5 | 500 | 3/3 (100%) | 27.3-33.7 | 29.7±2.3 | 3/3 (100%) | 21.2-25.4 | 23.4±1.6 |
| 7.0 | 1000 | 3/3 (100%) | 28.3-31.2 | 29.7±1.1 | 3/3 (100%) | 24.6-27.8 | 25.9±1.2 |
| 14.0 | 2000 | 3/3 (100%) | 30-36.6 | 33.6±2.5 | 3/3 (100%) | 24.6-25.7 | 25.3±0.3 |
| 63.0 | 9000 | 3/3 (100%) | 36.3-39.4 | 37.5±1.2 | 3/3 (100%) | 34.9-35.5 | 35.2±0.3 |
| 140.0 | 20000 | 3/5 (60%) | 35.2-39.0 | 36.5±2.0 | 3.5/5 (70%) | 29.8-35.4 | 31.9±1.9 |

**S4 Table.** Real-time PCR detection of low frequency spiked-in fruit flies (one *Bactrocera jarvisi* and five *Zeugodacus* cucumis) in bulk samples of varying sizes (approximating 1000 to 20,000 fruit flies) extracted using the optimised HotSOAK method (10ml of Buffer 1 was added/ ~3.5 g of flies and lysed at 75 °C for 10 minutes). Crude lysates and DNA extracts were tested in the species-specific real-time PCRs in triplicate for *Z. cucumis* and *B. jarvisi* real-time PCR (Li et al., 2019) (see S1 – Supplemental Methods).
