# Supplementary material for: Development of a cost-effective, morphology-preserving method for DNA isolation from bulk invertebrate trap catches: Tephritid fruit flies as an exemplar
Source: PLoS One. 2023 Feb 15;18(2):e0281759. doi: 10.1371/journal.pone.0281759 (PMC9931127; doi:10.1371/journal.pone.0281759)
Supplement: S1 File — (DOCX) [file pone.0281759.s005.docx]

**S1- Supplemental Methods**

The 18S real-time PCR reaction mixture of 20uL was prepared in a biosafety cabinet class 2 and contained 10.0 μL 2x PerfeCTa qPCR ToughMix (Quantabio) or 2x GoTaq Probe qPCR mastermix (Promega), 1.0 μL 20x Eukaryotic 18S rRNA Endogenous Control VIC^TM^/MGB probe, primer limited (Applied Biosystems^TM^, Thermo Fisher Scientific, Australia), 1.0 μL DNA or lysate and 8.0 μL nuclease-free water. Cycling conditions were 2 minutes at 50 °C and 10 minutes at 95 °C, followed by 35 cycles of 95 °C for 15 seconds and 60 °C for 1 minute acquiring to the yellow (VIC) channel on a Rotor-Gene RGQ Real-time PCR cycler (QIAGEN) with threshold set to 0.05 or QuantStudioTM 3 Real time PCR system (Thermo Fisher Scientific).

The *Bactrocera tryoni* and *B. jarvisi* COI real-time PCR assays were performed using published primers and probes [40-42]. PCR mixtures contained 12.5 μL 2X GoTaq Probe qPCR Master Mix (Promega) or 2x PerfeCTa qPCR ToughMix (Quantabio), 0.5 μM (Btry) or 0.2 μM (Bjarv) of each forward and reverse primers, 0.2 μM of probe, 5 μL of DNA or lysate, 4.5 μL nuclease-free water. Cycling conditions were: 2 minutes at 50 °C and 10 minutes at 95 °C, followed by 35 (40) cycles of 95 °C for 10 s and 58 °C for 1 minute acquiring to the green (FAM) channel on a Rotor-Gene RGQ Real-time PCR cycler (QIAGEN) with threshold set to 0.02 or QuantStudioTM 3 Real time PCR system (Thermo Fisher Scientific).

*B. tryoni* LAMP assays were performed on a Genie III (OptiGene) using published primers and conditions [40]. Data was analysed using the Optigene Genie Explorer Software Version 2.0.6.3 with the threshold set to 0.1.
